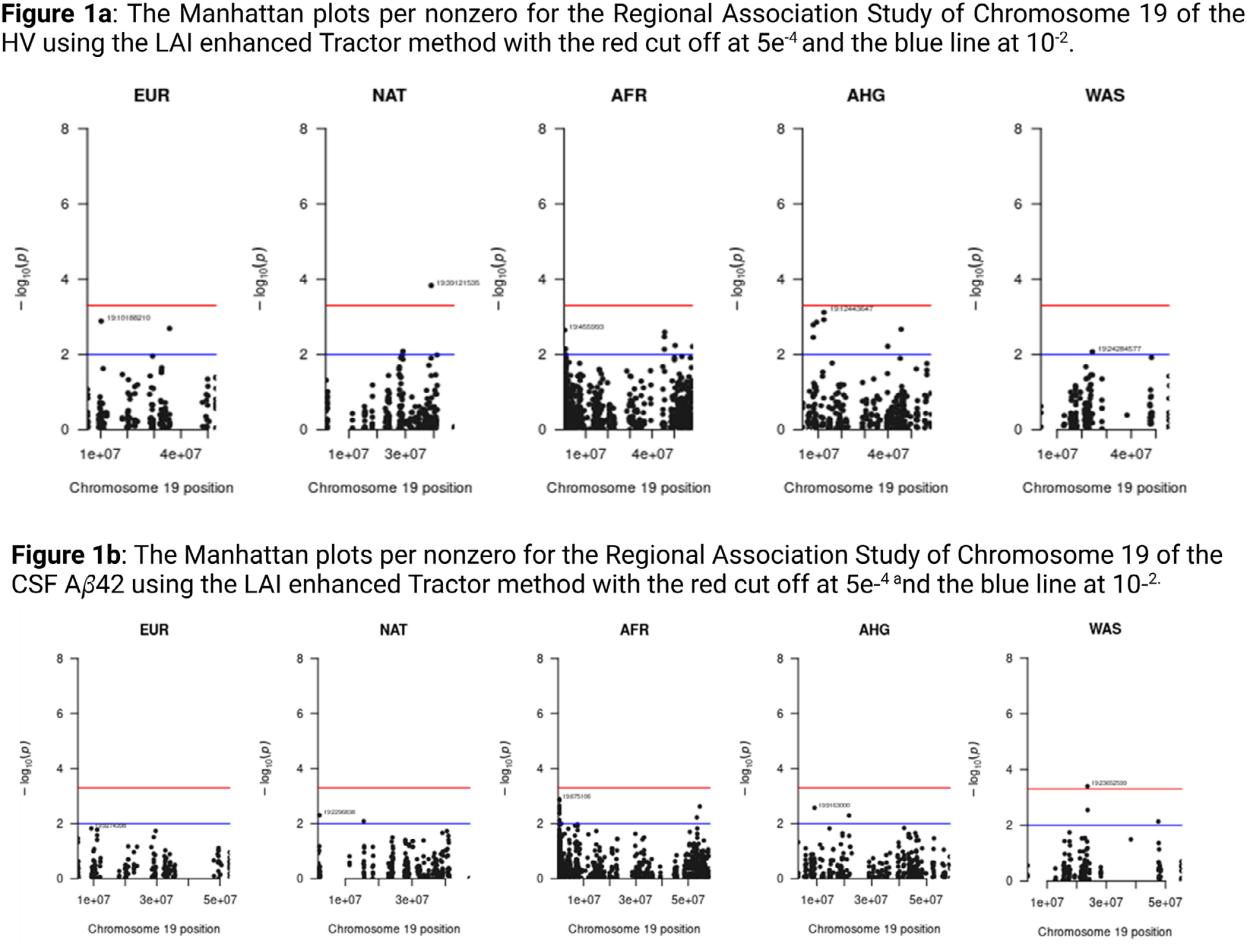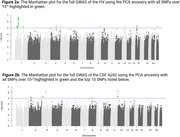# Integrating chromosome 19 local ancestry in genetic‐regional association studies reveal novel ancestry‐specific variants associated with hippocampal volume and CSF‐Aβ42

**DOI:** 10.1002/alz70856_105451

**Published:** 2026-01-07

**Authors:** Tavia E Evans, Elizabeth Chudleigh, Patricia Genius, Blanca Rodríguez‐Fernández, Federica Anastasi, Carolina Minguillón, Manel Esteller, Arcadi Navarro, Hieab H.H. Adams, Natalia Vilor‐Tejedor

**Affiliations:** ^1^ Radboud University Medical Center, Nijmegen, Netherlands; ^2^ Barcelonaβeta Brain Research Center (BBRC), Pasqual Maragall Foundation, Barcelona, Spain; ^3^ GBHI, Trinity College Dublin Ireland, Dublin, Dublin, Ireland; ^4^ Hospital del Mar Research Institute, Barcelona, Spain; ^5^ Centre for Genomic Regulation (CRG), Barcelona Institute of Science and Technology (BIST), Barcelona, Spain; ^6^ Hospital del Mar Research Institute (IMIM), Barcelona, Spain; ^7^ Instituto de Salud Carlos III, Madrid, Spain; ^8^ Institució Catalana de Recerca i Estudis Avançats (ICREA), Barcelona, Spain; ^9^ Josep Carreras Leukaemia Research Institute (IJC), Badalona, Barcelona, Spain; ^10^ Physiological Sciences Department, School of Medicine and Health Sciences, University of Barcelona (UB), Barcelona, Catalonia, Spain; ^11^ Institute of Evolutionary Biology (CSIC‐UPF) Universitat Pompeu Fabra, Barcelona, Spain; ^12^ BarcelonaBeta Brain Research Center (BBRC), Barcelona, Spain; ^13^ Latin American Brain Health (BrainLat), Universidad Adolfo Ibáñez, Santiago, Santiago, Chile; ^14^ Barcelonaβeta Brain Research Center (BBRC), Barcelona, Spain

## Abstract

**Background:**

Understanding the influence of genetic ancestry on Alzheimer's disease (AD) is essential to addressing health disparities and improving the generalizability of genetic discoveries. While most Genome‐Wide Association Studies (GWAS) rely on global ancestry, local ancestry inference (LAI) provides a refined methodology to detect ancestry‐specific genetic effects. We aimed to integrate local ancestry inference at chromosome 19 into regional genetic association analysis to identify local ancestry specific variants associated with hippocampal volume (HV) and cerebrospinal fluid (CSF) Amyloid beta 42 (Aβ42) levels.

**Methods:**

The framework integrated Gnomix tool for chromosome 19 local ancestry deconvolution and Tractor for regional ancestry‐informed GWAS adjusted by chronological age, sex, years of education, *APOE‐*ε4 status and eight components derived from LAI analysis, capturing the ancestry‐specific genetic structure. The analyses were performed using genetically well characterized participants from the ALFA cohort, focusing on two key AD‐related endophenotypes: HV (*N* = 1,325) and CSF‐Aβ42 levels (*N* = 282). Scans were obtained using a 3T Philips Ingenia CX MRI scanner using a uniform high‐resolution 3D protocol. CSF‐Aβ42 concentration was measured using the Elecsys® electrochemiluminescence immunoassay on the fully automated cobas e601 analyzer (Roche Diagnostics International Ltd.) at the Clinical Neurochemistry Laboratory, University of Gothenburg, Sweden. Genotyping was performed using the Illumina Infinium Neuro Consortium Array (build GRCh37/hg19), with imputation via the TOPMed Imputation Server using the r3 reference panel and Eagle v2.4 phasing.

**Results:**

LAI analysis revealed unique single nucleotide polymorphisms (SNPs) associated with HV and CSF‐Aβ42 that were undetected by traditional GWAS, highlighting the role of ancestry‐specific variants. Notably, significant SNPs in the *EIF3K* and *ZNF675* regions were linked to African (HV) and West Asian (Aβ42) ancestries [Figure 1], respectively, while classic GWAS and global ancestry models failed to identify these loci [Figure 2]. The LAI approach also provided enhanced resolution in identifying SNPs within the *APOE* region, emphasizing its relevance in understanding cross‐ancestry genetic risk.

**Conclusion:**

This study advances the understanding of genetic variation in AD. The findings support the inclusion of local ancestry analysis as a critical component of future AD genetic research, promoting equity in genetic discovery and personalized medicine.